# Targeted dual base editing with *Campylobacter jejuni* Cas9 by single AAV-mediated delivery

**DOI:** 10.1038/s12276-023-00938-w

**Published:** 2023-02-01

**Authors:** Jiyeon Kweon, An-Hee Jang, Eunji Kwon, Ungi Kim, Ha Rim Shin, Jieun See, Gayoung Jang, Chaeyeon Lee, Taeyoung Koo, Seokjoong Kim, Yongsub Kim

**Affiliations:** 1 Department of Biomedical Sciences, Asan Medical Institute of Convergence Science and Technology, Asan Medical Center, University of Ulsan College of Medicine, Seoul, 05505 Republic of Korea; 2 Stem Cell Immunomodulation Research Center, University of Ulsan College of Medicine, Seoul, 05505 Republic of Korea; 3 Department of Fundamental Pharmaceutical Sciences, Kyung Hee University, Seoul, 02447 Republic of Korea; 4 Department of Biomedical and Pharmaceutical Sciences, Kyung Hee University, Seoul, 02447 Republic of Korea; 5 Toolgen, Inc., Seoul, 08501 Republic of Korea

**Keywords:** Genetic engineering, Gene therapy

## Abstract

Various CRISPR‒Cas9 orthologs are used in genome engineering. One of the smallest Cas9 orthologs is cjCas9 derived from *Campylobacter jejuni*, which is a highly specific genome editing tool. Here, we developed cjCas9-based base editors including a cytosine base editor (cjCBEmax) and an adenine base editor (cjABE8e) that can successfully induce endogenous base substitutions by up to 91.2% at the *HPD* gene in HEK293T cells. Analysis of the base editing efficiency of 13 endogenous target sites showed that the active windows of cjCBEmax and cjABE8e are wider than those of spCas9-based base editors and that their specificities are slightly lower than that of cjCas9. Importantly, engineered cjCas9 and gRNA scaffolds can improve the base editing efficiency of cjABE8e by up to 6.4-fold at the *HIF1A* gene in HEK293T cells. Due to its small size, cjABE8e can be packaged in a single adeno-associated virus vector with two tandem arrays of gRNAs, and the delivery of the resulting AAV could introduce base substitutions at endogenous *ANGPT2* and *HPD* target sites. Overall, our findings have expanded the potential of the use of base editors for in vivo or ex vivo therapeutic approaches.

## Genome editing: Novel editors for CRISPR-Cas9 technology

Tiny genome editors that can fit inside a single delivery system could potentially improve the efficiency and specificity of the editing technology known as CRISPR-Cas9. This technology is used to edit parts of the genome and shows promise for disease therapeutics. Cas9 is a bacterial enzyme that acts as molecular scissors to edit DNA and is guided to the correct DNA region by specific RNA segments (gRNAs). Yongsub Kim at the University of Ulsan in Seoul, South Korea, and co-workers used one of the smallest known Cas9 enzymes, sourced from *Campylobactor jejuni*, to create two ‘base editors’ (BEs), capable of specific base substitutions. Results suggest the BEs are highly efficient, covering more potential targets than previous Cas9-based BEs. One of the BEs can be packaged, alongside two gRNAs, into a single viral vector for targeted delivery.

## Introduction

CRISPR–Cas9 is a powerful tool for genome engineering, and *Streptococcus pyogenes* Cas9 (spCas9) is the most widely used Cas9 for genome editing in various living systems. However, the large size of the spCas9 protein poses a barrier for in vivo or ex vivo delivery^1^. Aside from spCas9, many other Cas9 orthologs and their guide RNAs (gRNAs) have been identified^2–7^, of which *Campylobacter jejuni* Cas9 (cjCas9) is one of the smallest Cas9 orthologs. cjCas9 consists of 984 amino acid residues, which is significantly less than the 1384 amino acid residues of spCas9^8^. cjCas9 has several distinct characteristics compared with spCas9; for example, cjCas9 recognizes a 5′-NNNNRYAC-3′ PAM sequence, whereas spCas9 recognizes a 5′-NGG-3′ PAM sequence. Crystal structure analysis showed that cjCas9 can interact with both the target and nontarget strands of the PAM sequence, while spCas9 recognizes the PAM sequence through a nontarget strand^9^. In addition, cjCas9 induces indel mutations in human cells with longer gRNAs compared with those of spCas9, as the lengths of gRNAs for cjCas9 and spCas9 are 22 nt and 20 nt, respectively^8^.

Recently, several groups developed cjCas9-based genome engineering toolkits. Zhang et al. generated cjCas9-based transcription activators (termed miniCAFE) and showed that miniCAFE can activate target genes in human cells and *Caenorhabditis elegans*^10^. Zhang et al. also showed that miniCAFE can activate an endogenous gene in vivo through all-in-one adeno-associated virus (AAV) delivery. Because of the small size of cjCas9, cjCas9 has an advantage over spCas9, especially in an AAV delivery system.

CRISPR-mediated base editors (BEs), which have deaminase enzymes fused to Cas9-nickase, are powerful tools for the functional assessment of point mutations, which represent the largest class of pathogenic mutations^11^. To date, cytosine base editors (CBEs) and adenine base editors (ABEs) have been developed^12–14^, and improved versions of CBEs and ABEs have been reported to have enhanced activity and specificity^15–19^. Recent studies have shown that cjCas9 can also be used as a BE. Li et al. generated cjABE by fusing cjCas9-D8A with *Escherichia coli (E.coli)*-derived evolved-tRNA adenine deaminase (Tad A, ABE7.10 version) and successfully corrected the *TERT* promoter mutation by AAV infection^20^. Nakagawa et al. developed cjCas9-AID by fusing cjCas9-D8A with *Petromyzon marinus* cytidine deaminase 1 (PmCDA1) and showed that engineered cjCas9 (encjCas9, L58Y/D900K variants)-based cjCBE can induce targeted base editing in human cells, whereas wild-type cjCas9-based cjCBE cannot introduce endogenous base substitutions^21^. Although the studies by Li et al. and Nakagawa et al. showed that cjCas9 could be used in BEs, their characteristics were not analyzed in detail, and further studies are needed to define their functional characterization.

In this study, we developed cjCBEmax (cjCas9-D8A fused with APOBEC1 from BE4max), which induces a C:G to T:A conversion, and cjABE8e (cjCas9-D8A fused with evolved-TadA from ABE8e), which induces an A:T to G:C conversion. Using cjCBEmax and cjABE8e, we induced base substitutions at 13 endogenous target sites with high frequency and characterized their active windows and context dependency. Subsequently, we also applied encjCas9 and an engineered gRNA scaffold (e-scaffold) to improve base editing efficiencies, successfully constructed an all-in-one AAV vector containing cjABE8e and dual gRNAs, and induced base substitutions by AAV delivery.

## Materials and methods

### Plasmid constructs

pRGEN-CMV-CjCas9 (#89752; Addgene, Watertown, MA, USA) was used for pCMV-cjCas9 transfection, and pU6-Cj-sgRNA (Addgene #89753) was used to generate the gRNAs of cjCas9. The target sequences of gRNAs are provided in Supplementary Table 1. pCMV-cjCas9-D8A was generated by inducing a point mutation in the pRGEN-CMV-CjCas9 vector. The engineered *E. coli-*tRNA adenosine deaminase domain was amplified from ABE8e (Addgene #138489) and cloned into pCMV-cjCas9-D8A to generate a pCMV-cjABE8e vector. The APOBEC1 cytidine deaminase domain and UGI domains from pCMV-BE4MAX-3xHA (Addgene #112096) were amplified and cloned into pCMV-cjCas9-D8A to generate pCMV-cjCBEmax. pAAV-EFS-cjCas9-eEGFP-HIFIa (Addgene #137929) was modified to generate pAAV-cjABE8e-ANGPT2-HPD-2. The constructs pCMV-cjABE8e, pCMV-cjCBEmax, and pAAV-cjABE8e-2xU6 (gRNA empty vector of AAV vectors) will be available in Addgene.

### Cell culture and analysis of mutation frequency

HEK293T (ATCC CRL-3216) cells were maintained in Dulbecco’s Modified Eagle’s Medium (DMEM) with 10% fetal bovine serum (FBS) and 1% penicillin–streptomycin at 37 °C in a 0.05% CO_2_ atmosphere. Mycoplasma detection tests were performed every 2 weeks to confirm the absence of mycoplasma contamination. One day before transfection, 2.5 × 10^4^ HEK293T cells were seeded onto 96-multiwell plates, and transfection was performed using Lipofectamine 2000 (Thermo Fisher Scientific, Waltham, MA, USA) according to the manufacturer’s protocol. Briefly, 250 ng of plasmids (125 ng of gRNAs, 125 ng of pCMV-cjCas9 variants) were transfected into 60% confluent HEK293T cells, and genomic DNA was extracted using cell lysis buffer (0.05% SDS in pH 7.5 of 100 nM Tris-HCl and 100 μg/ml proteinase K) 72 h after transfection. The cell lysate was stored at −20 °C until use. To measure the mutation frequencies of each sample, genomic DNA was amplified with the target-specific primer pairs listed in Supplementary Table 1, and NGS libraries were further generated with TruSeq HT Dual Index primer pairs. The NGS libraries were subjected to paired-end sequencing using Mini-seq or iSEQ (Illumina, San Diego, CA, USA), and data were analyzed using MAUND as previously described^22^.

### Production and titration of the AAV vector

HEK293T cells were transfected with pAAV-cjABE8e-gRNA-ANGPT2-HPD-2, pAAV-DJ encoding AAV2rep, AAV-DJcap, and helper plasmids; cells were maintained in DMEM with 2% FBS. Recombinant pseudotyped AAV vector stock was generated by PEI coprecipitation using PEI-MAX (Polysciences, Warrington, PA, USA), and transfection was conducted with three plasmids at a molar ratio of 1:1:1. After 72 h of incubation, the cells were lysed, and AAV particles were purified by iodixanol step-gradient ultracentrifugation. The number of vector genomes was determined by quantitative PCR.

### AAV transduction in cells

HEK293T cells were infected with AAV particles at different viral genome (vg)/cell multiplicities of 1 × 10^2^ vg/cell, 1 × 10^3^ vg/cell, and 1 × 10^4^ vg/cell, determined by quantitative PCR, and maintained in DMEM with 2% FBS. After 72 h of incubation, the infected cells were harvested and subjected to targeted deep sequencing.

### Western blot assay

Three plasmids—pRGEN-CMV-CjCas9, pCMV-cjABE8e, and pCMV-cjCBEmax—were transfected into HEK293T cells, and whole-cell lysates (WCLs) were prepared using 1 × RIPA lysis buffer 72 h after transfection and quantified by Bradford assay. Western blot assays were conducted using the iWestern system (Thermo Fisher Scientific) according to the manufacturer’s protocol. Briefly, 10 μg of WCLs were separated in 4–12% bis-tris gradient gels and transferred to a polyvinylidene difluoride membrane using the iBlot 2 Dry Blotting System. Immunoblotting was conducted with anti-HA (#9110; Abcam, Cambridge, UK) and anti-glyceraldehyde 3-phosphate dehydrogenase (anti-GAPDH, sc-47724; Santa Cruz Biotechnology, Dallas, TX, USA) antibodies. The immunoblotted proteins were detected by enhanced chemiluminescence.

### Statistics and reproducibility

All experiments were performed in biologically independent triplicates, and the data are reported as the mean ± standard error of the mean and plotted using GraphPad Prism (GraphPad Software, La Jolla, CA, USA).

## Results

### Construction of cjCBEmax and cjABE8e

**Fig. 1:**
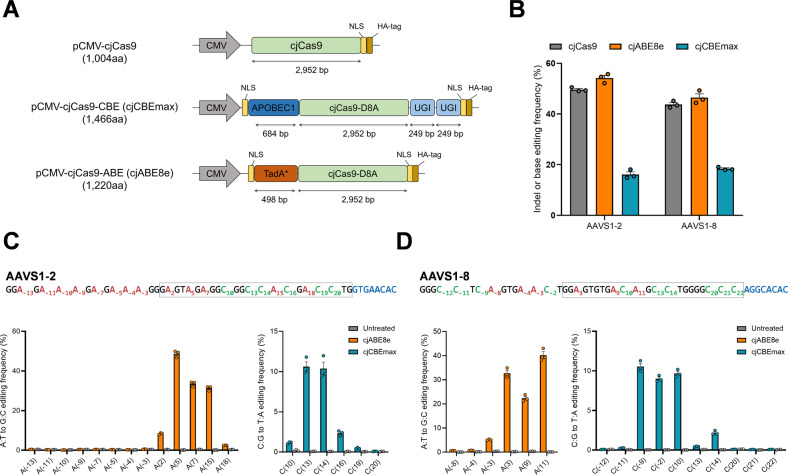
*Campylobacter jejuni* Cas9-mediated cytosine and adenine base editing in human cells. **A** Schematic overviews of the constructs of cjCas9, cjABE8e, and cjCBEmax. cjCBE and cjABE consist of 1466 and 1220 amino acid residues, respectively. **B** Mutation frequencies of cjCas9, cjABE, and cjCBE at the endogenous AAVS1-2 and AAVS1-8 target sites in HEK293T cells. Base editing frequency of individual adenine and cytosine bases within a 37-nt window, 22-nt of spacer, and 15-nt outside the spacer sequence, around the (**C**) AAVS1-2 target site and the (**D**) AAVS1-8 site. Within the 37-nt window, adenine and cytosine are highlighted in red and green, respectively, and the spacer sequences and PAM of gRNAs are indicated in blue boxes, respectively. All experiments were conducted in biologically independent triplicates. Error bars indicate the standard error of the mean.

We used improved variants of spCas9-based BEs—BE4MAX and ABE8e—to generate highly active cjCas9-based BEs—cjCBEmax and cjABE8e. Two improved base editors (BE4MAX and ABE8e) were developed and reported in the literature^15,19^; BE4MAX contains codon-optimized APOBEC1 cytidine deaminase and UGI domains, and ABE8e has an evolved TadA deoxyadenosine deaminase. We cloned these domains into the pCMV-cjCas9-D8A construct to generate cjCBEmax and cjABE8e (Fig. 1A). Compared with the original spCas9-based BEs, the coding sequences of cjCBEmax and cjABE8e were approximately 1.3-fold smaller, and their expression levels were confirmed by Western blot assay (Supplementary Fig. 1). We transfected these constructs into HEK293T cells with AAVS1-2 and AAVS1-8 gRNA, which was shown to allow cjCas9 to induce indels with a high mutation frequency^8^, and we analyzed the mutation frequencies by targeted deep sequencing.

The conventional active window of base editors was 4–9 positions in their spacer sequences; however, because we did not know the active window of the cjCas9-based BEs, we analyzed base editing frequencies over a broader range, including 15 nt outside the spacer sequence. At the AAVS1-2 and AAVS1-8 target sites, cjABE8e induced A:T to G:C conversions by up to 55.7 and 49.3%, and cjCBEmax induced C:G to T:A conversions by up to 18.1 and 19.1%, respectively (Fig. 1B). Interestingly, both cjCBEmax and cjABE8e were able to introduce base substitutions outside the conventional active windows (Fig. 1C, D). cjABE8e induced an A:T to G:C conversion by up to 32.0% at the A(15) position of AAVS1-2 target sites and 43.0% at the A(11) position of AAVS1-8 target sites.

Although there was no cytosine in the conventional active window, cjCBEmax could induce base C:G to T:A conversions in both AAVS1-2 and AAVS1-8 target sites, especially at the C(−9) position of the AAVS1-8 target site (up to 11.3%). At these two sites, we found that cjABE8e could not efficiently edit adenines located outside the spacer sequence, whereas cjCBEmax could convert cytosines located outside the spacer sequence. Taken together, we demonstrated that cjCBEmax and cjABE8e can induce base conversion at endogenous target sites, suggesting that their active window might be much wider than that of conventional BEs.

### Characterization of cjCBEmax and cjABE8e

**Fig. 2:**
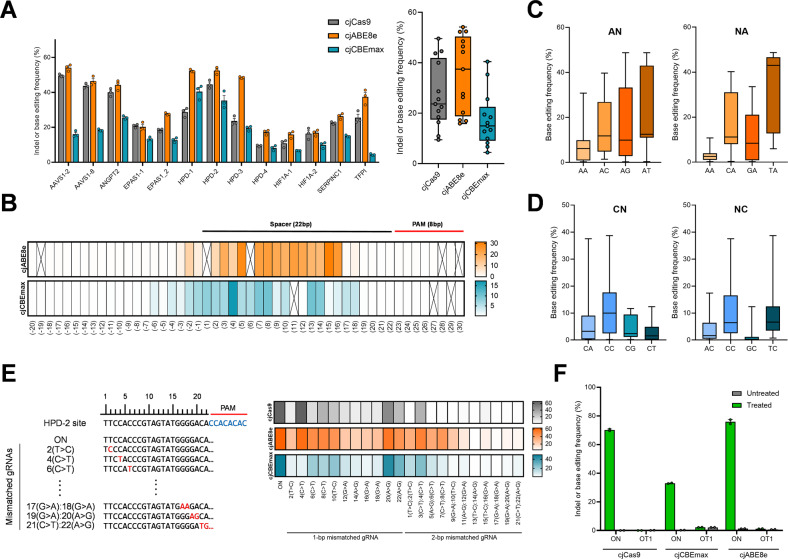
Functional characterization of cjCBE and cjABE at endogenous target sites in human cells. **A** Bar graphs showing the mutation frequencies of each endogenous target site. The mutation frequencies of cjCas9, cjABE, and cjCBE across the 13 target sites are summarized in the right panel. **B** Heatmap of the base editing activity of cjABE and cjCBE within the 50-nt window across 13 endogenous target sites. The position of each nucleotide was numbered relative to the starting position of the target spacer sequences. A box with an X means there was no adenine or cytosine at those positions. Analysis of the sequence contexts around (**C**) the target adenine of cjABE and (**D**) the target cytosine of cjCBE. **E** The target site with PAM sequences of HPD-2 gRNA is shown in the left panel. The position of each nucleotide was designated relative to the starting position of the target spacer sequences. Mutation frequencies of cjCas9, cjABE, and cjCBE with mismatched gRNAs are shown in the heatmap in the right panel. ON means HPD-2 gRNAs with no mismatch to target sites. The numbers listed below the heatmap indicate the positions of the mismatches in each gRNA. **F** Analysis of potential off-target mutations of AAVS1-8 gRNAs with cjCas9, cjABE, and cjCBE. The target sequences of each site are listed in Supplementary Table 1. All experiments were conducted in biologically independent triplicates. Error bars indicate the standard error of the mean.

To further characterize cjCBEmax and cjABE8e, we cloned gRNAs to edit 11 additional target sites and transfected them with cjCas9, cjCBEmax, and cjABE8e in HEK293T cells. As shown in Fig. 2A, cjCBEmax induced C:G to T:A conversions by up to 43.5% at the *HPD*-1 target site, and cjABE8e induced A:T to G:C conversions by up to 54.3% at the *HPD*-2 target site. We found that cjABE8e had higher activity than cjCBEmax at all target sites, with cjABE8e and cjCBEmax showing average mutation frequencies of 35.4 and 17.4%, respectively, across 13 endogenous target sites. To characterize the base editing active window of cjCBEmax and cjABE8e, we analyzed the substitution frequencies of individual cytosines and adenines in a 50-nt window (Fig. 2B and Supplementary Fig. 2). In line with the results for AAVS1-2 and AAVS1-8 (Fig. 1C, D), we found that cjCBEmax and cjABE8e had a wider active window than spCas9-based BEs. cjCBEmax could edit the C(−12) position by up to 1.9% in the *EPAS1*-1 target site and the C(19) position by up to 0.7% in the *HIF1A*-1 target site. Particularly in the *SERPINC1* target site, cjCBEmax induced base substitutions by up to 13.5% at the C(13) position, which was a much higher frequency than those of the C(8) position located in the conventional active window. Compared with cjCBEmax, cjABE8e showed a narrower active window across the 13 target sites, inducing base substitutions by up to 9.4% at the A(−3) position of the *ANGPT2* target site and 7.1% at the A(18) position of the *EPAS1*-1 target site.

Next, we examined whether the context around adenines and cytosines affected the base editing activities of cjCBEmax and cjABE8e. For cjABE8e, the AA sequence context had an adverse effect on the adenine base editing activity, while the TA sequence context tended to enhance the base editing activity (Fig. 2C). The cjCBEmax exhibited relatively high cytosine editing activity in the context of a TC sequence compared with that in the context of a GC sequence (Fig. 2D). Previously, Song et al. analyzed the correlation between the efficiency of spCas9-based BEs and sequence context^23^, and we found that cjCBEmax and cjABE8e showed similar trends to spCas9-based BEs. Overall, we demonstrated that cjCBEmax and cjABE8e can induce substitutions at various endogenous sites with a wider active window than that of spCas9-based BEs and that the sequence context affecting their activity was similar to that of spCas9-based BEs.

### Specificity of cjCBEmax and cjABE8e

Next, we assessed the tolerance of cjCBEmax and cjABE8e to mismatched gRNAs. A total of 22 gRNAs having one or two mismatches with the target sequences were constructed and transfected into HEK293T cells with cjCas9, cjCBEmax, and cjABE8e (Supplementary Table 1). We analyzed the indel and base substitution frequencies by targeted deep sequencing and compared the tolerance for mismatched gRNAs (Fig. 2E). In most cases, cjCBEmax and cjABE8e were more tolerant to base mismatches in the PAM-distal region than in the PAM-proximal region, whereas 20(A>G) and 22(A>G) mismatched gRNAs showed a different trend. For example, cjCBEmax showed 44.2% base editing activity with 20(A>G)-mismatched gRNA containing a 1 bp mismatch at position 20 of the spacer sequence, which was comparable to the base editing activity with HPD-2 gRNA. As the mismatch tolerance for 20(A>G) and 22(A>G), which had a 1 bp mismatch at the closest location in the PAM, was also observed with cjCas9, we speculated that this might be a characteristic of cjCas9 or a target-specific trait.

For most gRNAs with 2-bp mismatches, cjCas9 was not tolerant, whereas cjCBEmax and cjABE8e had modest tolerance that was proportional to the distance from the PAM region. Especially with 1(T>C):2(T>C)-mismatched gRNA containing 2 bp mismatches at positions 1 and 2, cjCBEmax and cjABE8e induced substitutions by up to 15.0 and 35.6%, respectively, whereas cjCas9 showed an indel frequency of 0.9%. These results suggest that cjCBEmax and cjABE8e have a slightly lower specificity than cjCas9.

We also sought to identify the endogenous off-target effects of cjCBEmax and cjABE8e. The potential off-target sites of each gRNA were analyzed in silico using Cas-OFFinder^24^, and we selected an AAVS1-8-OT1 potential off-target site containing a 2 bp mismatch in the target spacer sequence (Supplementary Table 2). We analyzed the endogenous mutations by targeted deep sequencing and found that cjCBEmax, cjABE8e, and cjCas9 showed no detectable endogenous off-target mutations (Fig. 2F).

### Improvement of cjCBEmax and cjABE8e

**Fig. 3:**
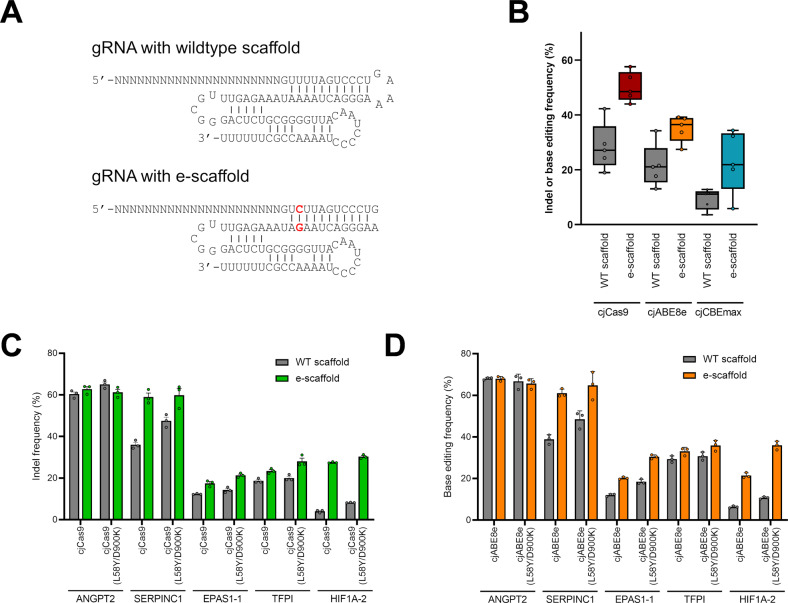
Engineering of gRNA scaffold and cjCas9 protein for improving base editing efficiency. **A** The structures of the gRNAs of cjCas9 predicted using the Mfold web server^42^. The e-scaffold was developed by U:A to C:G flip in the lower stem and ‘AA’ deletion in the tetraloop of the wild-type scaffold. **B** Mutation frequencies of cjCas9, cjABE, and cjCBE with gRNAs containing wild-type scaffold or e-scaffold sequences at five endogenous target sites. **C** Indel frequencies of cjCas9 and engineered cjCas9 (cjCas9-L58Y/D900K) with gRNAs containing wild-type scaffold or e-scaffold. **D** Base editing frequencies of cjABE and engineered cjABE (cjABE-L58Y/D900K) with gRNAs containing wild-type scaffolds or e-scaffolds. All experiments were conducted in biologically independent triplicates. Error bars indicate the standard error of the mean.

To improve the base editing efficiency of cjCBEmax and cjABE8e, we engineered the scaffold sequences of gRNAs according to previous studies regarding spCas9 gRNA engineering^25,26^. As shown in Fig. 3A, we removed a putative terminator motif of four consecutive uracils by a single A:U to G:C conversion to avoid premature termination of gRNA transcription, truncated the tetraloop to shorten the length of gRNAs and named the engineered scaffold the “e-scaffold”. We cloned gRNAs with e-scaffolds for five endogenous target sites and compared their mutation frequencies to those of wild-type scaffolds by targeted deep sequencing. As shown in Fig. 3B, the e-scaffold improved the mutation frequencies of cjCas9, cjCBEmax, and cjABE8e at all five target sites.

A recent study showed that an L58Y/D900K double mutation in cjCas9 (encjCas9) can improve the activity of cjCas9^21^. To determine whether the L58Y/D900K double mutation was synergetic with the e-scaffold, we first compared the indel frequency of cjCas9 and encjCas9 combinations with gRNAs with the wild-type scaffold or e-scaffold (Fig. 3C). We found that encjCas9 had improved activity compared with cjCas9 and had synergetic effects with the e-scaffold across five target sites; in particular, at the *HIF1A*-2 target site, the combination of encjCas9 and the e-scaffold enhanced the indel activities by 7.6-fold (from 4.0 to 30.3%). We then introduced the L58Y/D900K double mutation in cjABE8e (encjABE8e) and tested its activity with gRNAs bearing the e-scaffold (Fig. 3D). At five target sites, encjABE8e showed improved base editing activity compared with cjABE8e, which was synergetic with the e-scaffold but did not significantly change the base editing window (Supplementary Fig. 3).

### AAV vector of cjABE8e for base editing

**Fig. 4:**
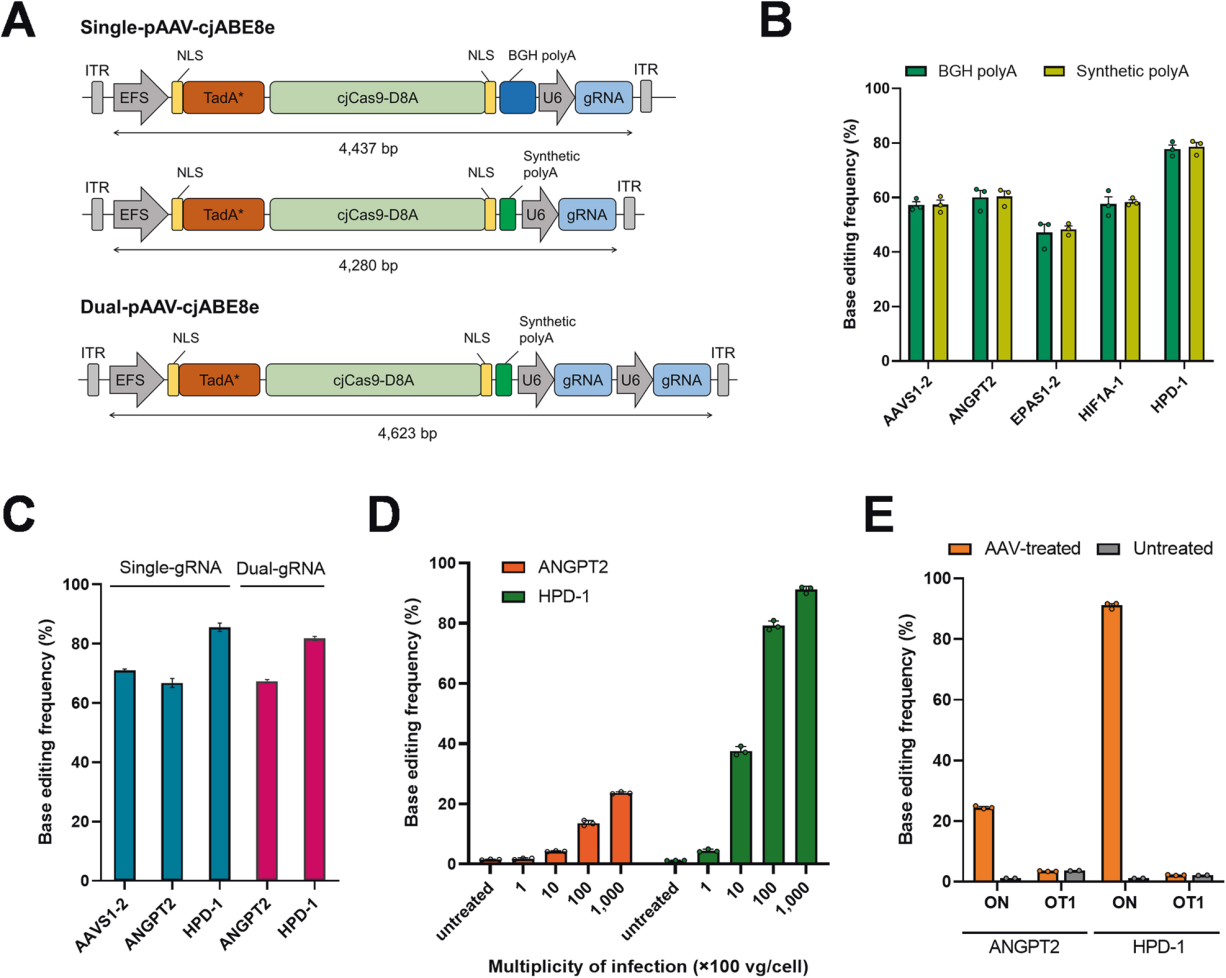
Adenine base editing using cjABE with two tandem arrays of gRNAs via single AAV delivery. **A** Schematic overviews of single-pAAV-cjABE and dual-pAAV-cjABE. The lengths between the two LTRs are shown. **B** Comparison of the base editing frequency of single-pAAV-cjABE containing a BGH polyA sequence or synthetic polyA sequences. **C** Comparison of the base editing frequency of single-pAAV-cjABE and dual-pAAV-cjABE with gRNAs containing synthetic polyA. **D** Base editing efficiency of dual-pAAV-cjABE at *ANGPT2* and *HPD*-1 target sites following infection with AAV particles at a multiplicity of different viral genome (vg)/cell ranging from 1 × 10^2^ to 1 × 10^5^ vg/cell determined by quantitative PCR. **E** Analysis of potential off-target mutations in dual-pAAV-cjABE-treated cells. All experiments were conducted in biologically independent triplicates. Error bars indicate the standard error of the mean.

We next examined whether cjABE8e could be packaged into an AAV vector. Because of their limited packaging capacity, spCas9-based BEs are challenging to deliver through a single AAV vector system. Since cjABE8e was small enough to package into an AAV vector, we speculated that cjABE8e and two tandem arrays of gRNA might be integrated into a single AAV vector (Fig. 4A). To further reduce the size of the construct, we investigated whether a previously known synthetic polyadenylation (polyA) sequence was compatible with cjABE8e. As the synthetic polyA sequence is 49 bp long, which is much shorter than the 225 bp bovine growth hormone (BGH) polyA sequence, it can provide more space for AAV packaging.

We first cloned single-pAAV-cjABE8e constructs containing BGH polyA or synthetic polyA sequences and transfected them into HEK293T cells (Fig. 4B). Targeted deep sequencing showed that single-pAAV-cjABE8e constructs with synthetic polyA and BGH polyA sequences had similar base editing frequencies across five target sites. We then constructed a dual-pAAV-cjABE8e vector containing two gRNAs targeting *ANGPT2* and *HPD*-1 and compared its base editing efficiency with those of single-pAAV-cjABE8e vectors. As a result, we found that the dual-pAAV-cjABE8e vector could induce base substitutions by 68.4 and 82.9% at the *ANGPT2* and *HPD*-1 target sites, respectively, which were comparable with those of single-pAAV-cjABE8e vectors (Fig. 4C). Subsequently, we produced AAV particles and infected them into HEK293T cells, and we found that the base editing frequencies accumulated in a dose-dependent manner up to 24.0 and 91.9% at the *ANGPT2* and *HPD*-1 target sites, respectively (Fig. 4D). We also investigated potential off-target sites of *ANGPT2* and *HPD*-1 in silico and measured mutations at these sites by targeted deep sequencing in AAV-infected HEK293T cells, but we did not find detectable off-target mutations (Fig. 4E and Supplementary Table 2).

## Discussion

In this study, we demonstrated that cjCas9-based BEs cjCBEmax and cjABE8e can induce nucleotide substitutions in the human genome with high efficiency and that their active window is much wider than that of spCas9-based BEs. We further improved the base editing activity of cjCBEmax and cjABE8e by applying engineered cjCas9 and a scaffold of gRNAs. Due to their small size, cjABE8e and two tandem arrays of gRNAs can successfully be packaged into a single AAV vector and form a powerful tool for efficient genome editing in vivo.

CRISPR-mediated BEs have emerged as powerful tools for therapeutic uses in human diseases, but in vivo delivery of BEs remains a major challenge due to the large size of BEs^1^. Recently, several groups showed that ABEs containing Cas9 orthologs that are smaller than spCas9, *Staphylococcus aureus* Cas9 (saCas9), *Staphylococcus Auricularis* Cas9 (SauriCas9) and *Neisseria meningitidis* Cas9 (Nme2Cas9) with their single gRNA can be embedded into a single AAV vector^27–29^. Compared with saCas9- and Nme2Cas9-ABE, cjABE8e is smaller in size, so there is more room for packaging other constructs in a single-AAV vector, such as one additional copy of gRNA. Using this construct, we successfully introduced base substitutions in multiple loci by single AAV delivery.

We found that cjCas9-based BEs have wider active windows than spCas9-based BEs, with cjABE8e being able to edit an adenine at positions (−3) to (+18) and cjCBEmax being able to edit a cytosine at positions (−6) to (+18). In particular, cjCas9-BEs can introduce base substitutions outside the target spacer sequences, which is likely because cjCas9 is small enough for deaminases to approach the target DNA. Similar to cjCas9-BEs, saCas9-BEs have a wider active window than spCas9-BEs^30,31^. The active window of cjCas9-based BEs can be made narrower for target-specific nucleotide substitution by using engineered deaminases or altering the linkers between cjCas9 and deaminase^16,30,32^; however, the feature of a wide active window may be an advantage for use in gene silencing, including the disruption of coding sequences and canonical splice sites without inducing double-strand breaks in the DNA^33–37^.

In addition to the editing window, cjCas9-mediated BEs might be further developed for precise base editing in the genome. We found that cjCas9-mediated BEs were tolerant to 1- or 2-bp mismatches in the PAM-distal region and 1 bp mismatches in the PAM-proximal region, a trend similar to that of spCas9-mediated base editors^38,39^. To improve the fidelity of cjCas9-mediated BEs, cjCas9 and gRNAs could be further engineered. Unwanted DNA or RNA deamination in a gRNA-independent manner is also a significant issue for precise base editing. To reduce unwanted off-target effects on the genome and transcriptome, engineered deaminase proteins that reduce unwanted DNA and RNA off-target effects can be used^16,17,40,41^.

Recent studies by Li et al.^20^ and Nakagawa et al.^21^ reported the generation of cjCas9-AID, cjCas9-D8A nickase fused with PmCDA1, cjCas9-based ABE (termed cjABE), and cjCas8-D8A nickase fused with TadA (ABE7.10 version). Although these studies showed that cjCas9 could be used as a BE, its characteristics, including its active window and context dependency, were not analyzed in detail. Nakagawa et al. showed that cjCas9-AID mostly failed to induce C:G to T:A conversions, so they developed encjCas9-AID, an encjCas9-D8A nickase fused with AID, for targeted cytidine base editing. encjCas9-AID, but not cjCas9-AID, showed base editing activity in human cells, whereas cjCBEmax containing wild-type cjCas9 showed high base editing activity in this study. Li et al. generated cjABE and corrected the −124 C>T *TERT* promoter mutation via AAV delivery in vivo. Compared with cjABE, cjABE8e used in this study has a much smaller size, enough to be packaged with dual gRNAs in AAV vectors, and showed higher base editing activity.

In summary, we successfully developed small base editors, cjCBEmax and cjABE8e, using cjCas9 and defined their functional characteristics, including active windows and context dependency. Recently, other types of minimal BEs (Cas12f- or TnpB-mediated base editors) have been developed, and we expect that these minimal BEs, including cjCas9-mediated base editors capable of delivery with a single AAV, will be used to broaden the usage of base editors in biomedical research.

## Supplementary information

Supplmentaray Information

## Data Availability

The sequencing data for this study are available from the Sequencing Read Archive (https://www.ncbi.nlm.nih.gov/sra) under accession number PRJNA850677.
