# Supplementary material for: Targeted dual base editing with Campylobacter jejuni Cas9 by single AAV-mediated delivery
Source: Exp Mol Med. 2023 Feb 1;55(2):377–84. doi: 10.1038/s12276-023-00938-w (PMC9981745; doi:10.1038/s12276-023-00938-w)
Supplement: Supplementary file 1 — Supplmentaray Information [file 12276_2023_938_MOESM1_ESM.pdf]

**Supplementary Information**

# Targeted dual base editing with Campylobacter jejuni Cas9 by single AAV-mediated delivery

Jiyeon Kweon[1, 2], An-Hee Jang[1, 2], Eunji Kwon[3,4], Ha Rim Shin[1, 2], Jieun See[1, 2], Gayoung Jang[1, 2], Chaeyeon Lee[1, 2], Taeyoung Koo[3,4], Yongsub Kim[1, 2, *]

[1] Department of Biomedical Sciences, Asan Medical Institute of Convergence Science and Technology, Asan Medical Center, University of Ulsan College of Medicine, Seoul 05505, Republic of Korea
[2] Stem Cell Immunomodulation Research Center, University of Ulsan College of Medicine, Seoul 05505, Republic of Korea
[3] Department of Fundamental Pharmaceutical Sciences, Kyung Hee University, Seoul, 02447, Republic of Korea
[4] Department of Biomedical and Pharmaceutical Sciences, Kyung Hee University, Seoul, 02447, Republic of Korea

* Corresponding author: Yongsub Kim, PhD (email: yongsub1.kim@gmail.com)

## Table of Contents

Supplementary Figure 1. Protein expression levels of cjCBEmax and cjABE8e.

Supplementary Figure 2. Base editing frequency of individual adenine and cytosine within a 50-nt window around target sites.

Supplementary Figure 3. Base editing frequency of individual adenine by cjCas9 (L58Y/D900K) and e-scaffold gRNAs.

Supplementary Table 1. List of target sequences and PCR primers used in this study.

Supplementary Table 2. List of potential off-target sites of cjABE target sites in the human genome.

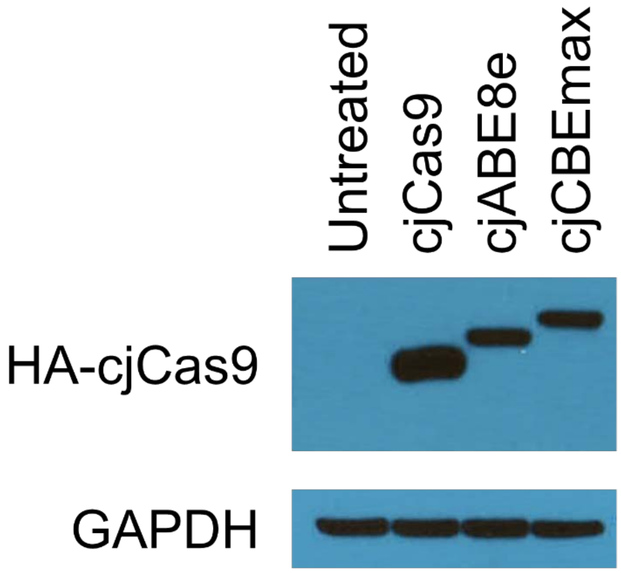


**Supplementary Fig. 1. Protein expression levels of cjCBEmax and cjABE8e.** Western blotting of HEK293T cells transfected with cjCas9, cjCBEmax, and cjABE8e. GAPDH served as the loading control.

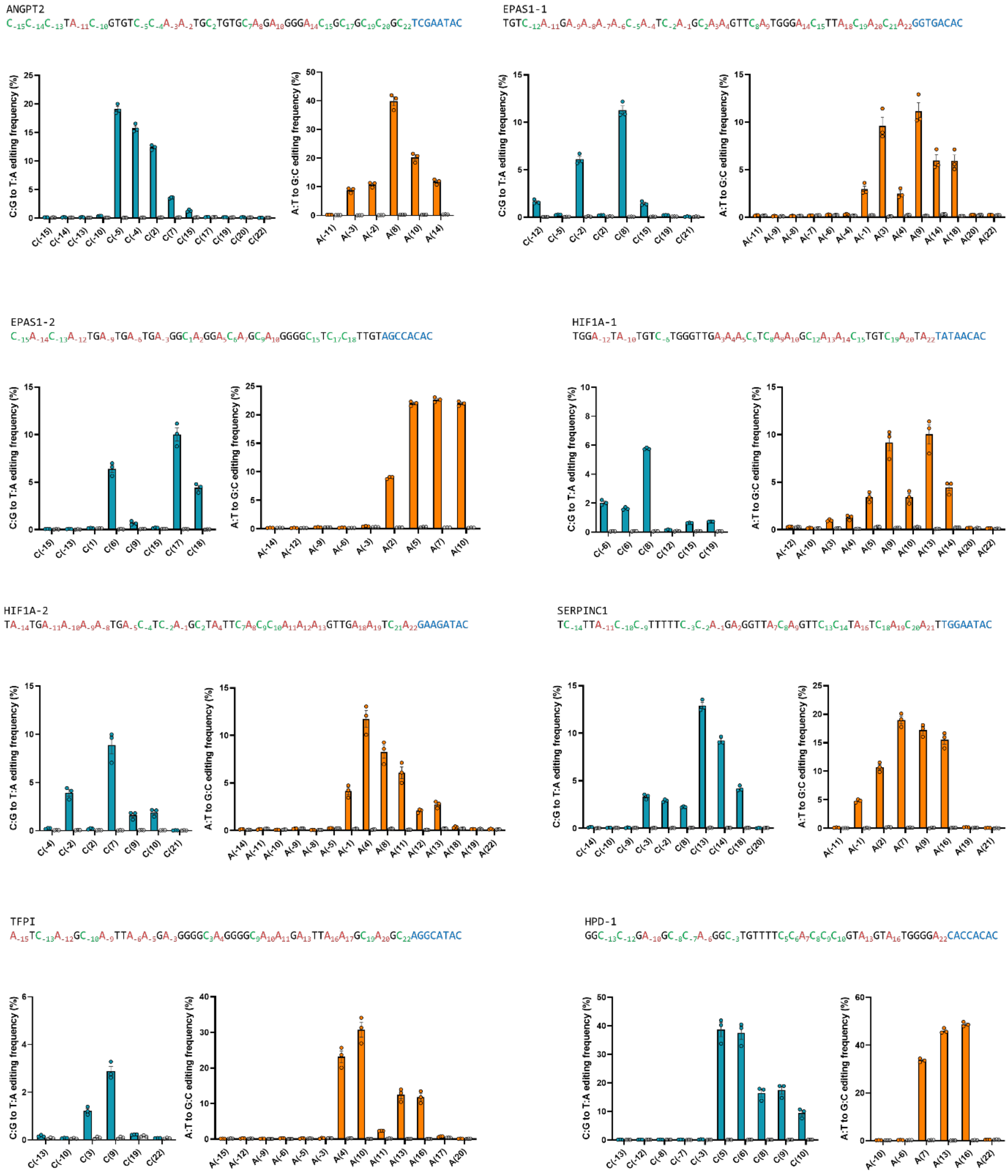

ANGPT2
EPAS1-1
EPAS1-2
HIF1A-1
HIF1A-2
SERPINC1
TFPI
HPD-1
C:G to T:A editing frequency (%)
A:T to G:C editing frequency (%)

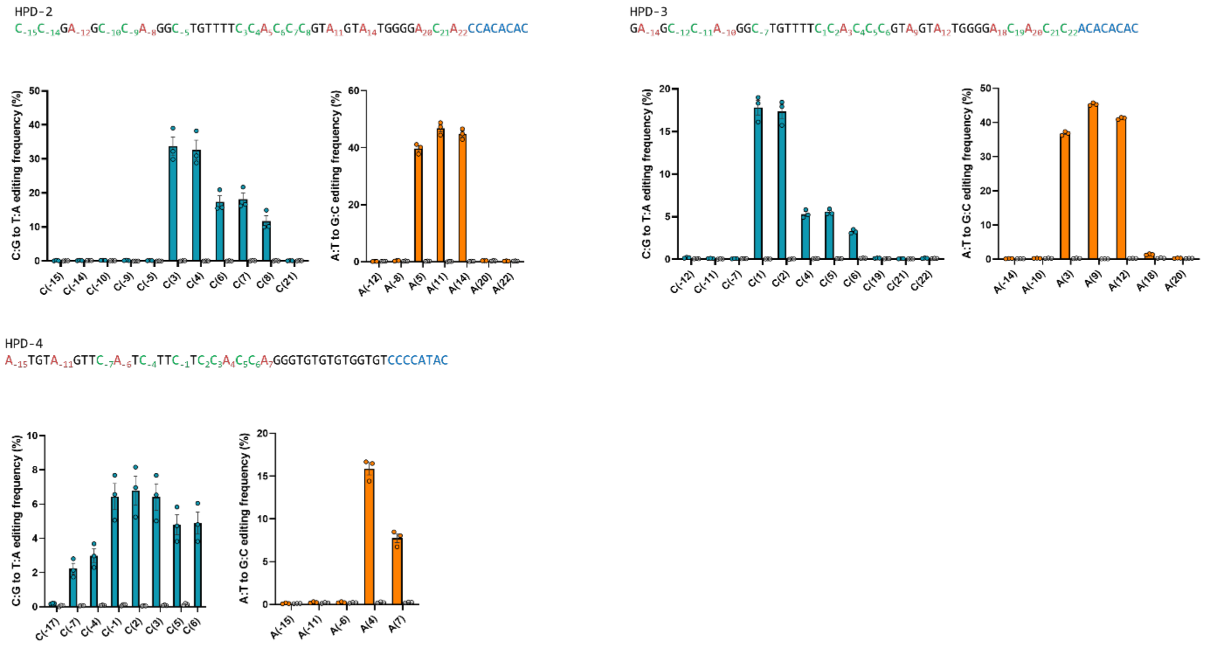


**Supplementary Fig. 2. Base editing frequency of individual adenine and cytosine within a 50-nt window around target sites.** Within the 50-nt window, adenine and cytosine are highlighted in red and green, respectively. The PAM sequences are highlighted in blue. The position of each nucleotide was numbered relative to the starting position of the target spacer sequences. All experiments were conducted in biologically independent triplicates. Error bars indicate standard error of the mean.

(a)

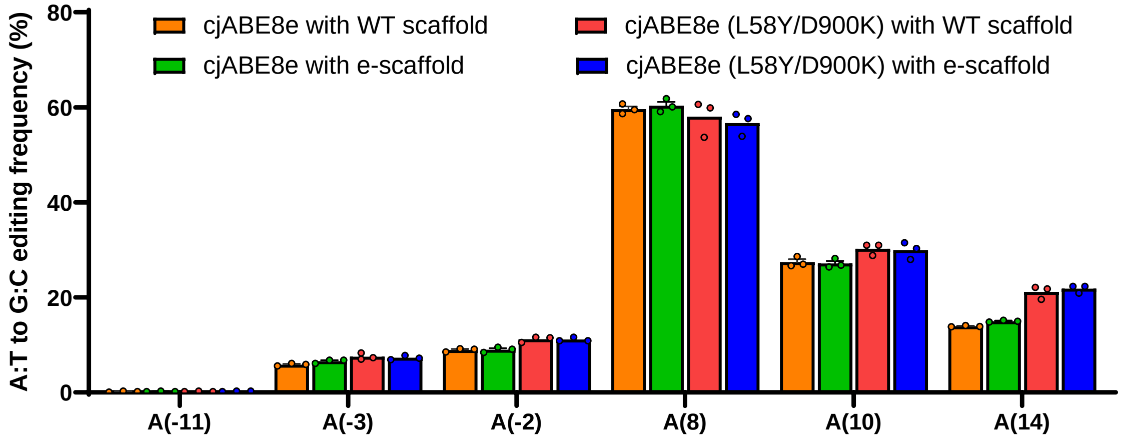
cjABE8e with WT scaffold
cjABE8e with e-scaffold
cjABE8e (L58Y/D900K) with WT scaffold
cjABE8e (L58Y/D900K) with e-scaffold
A:T to G:C editing frequency (%)
80
60
40
20
0
A(-11)
A(-3)
A(-2)
A(8)
A(10)
A(14)

(b)

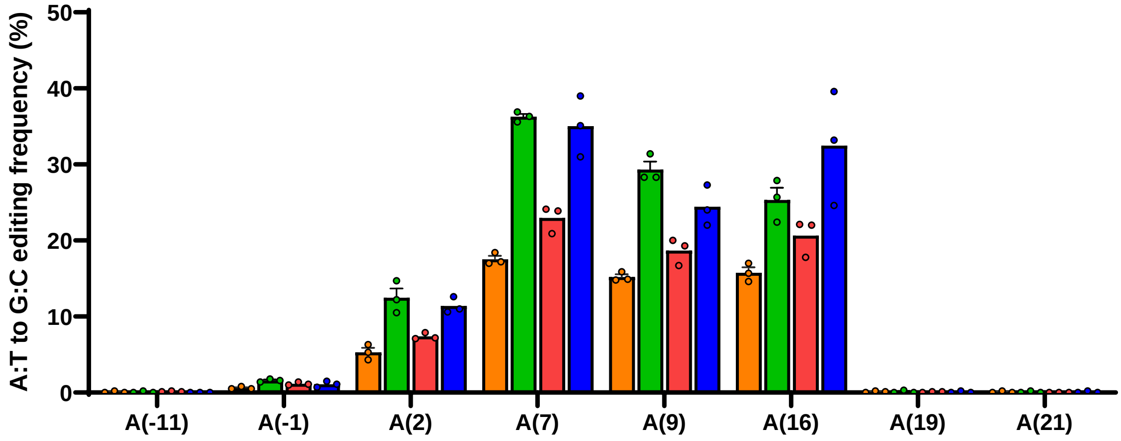
A:T to G:C editing frequency (%)
50
40
30
20
10
0
A(-11)
A(-1)
A(2)
A(7)
A(9)
A(16)
A(19)
A(21)

(c)

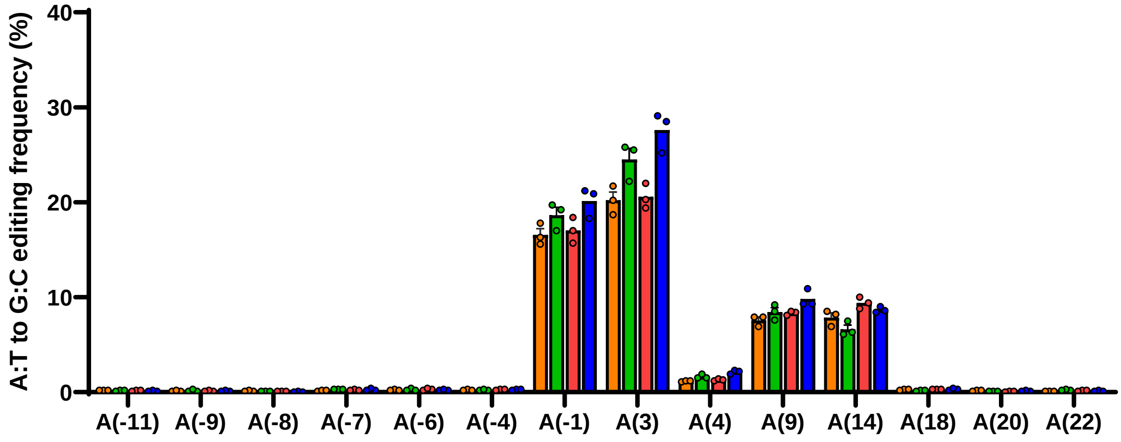
A:T to G:C editing frequency (%)
40
30
20
10
0
A(-11)
A(-9)
A(-8)
A(-7)
A(-6)
A(-4)
A(-1)
A(3)
A(4)
A(9)
A(14)
A(18)
A(20)
A(22)

(d)

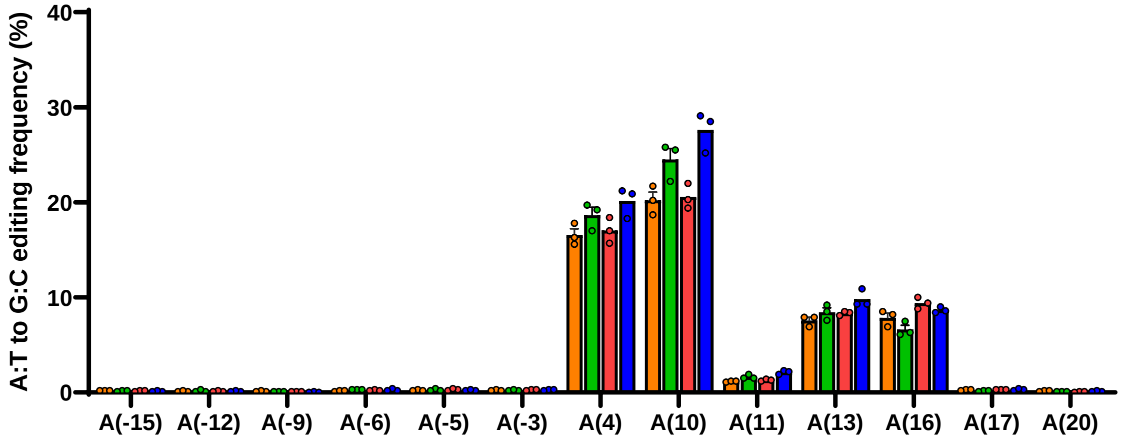


(e)

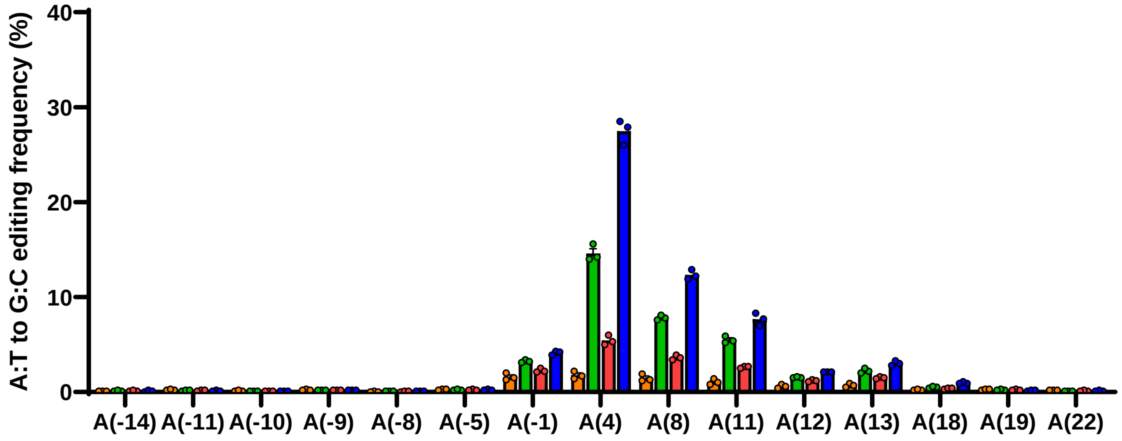


**Supplementary Fig. 3. Base editing frequency of individual adenine by cjCas9 (L58Y/D900K) and e-scaffold gRNAs.** The encjCas9 showed improved base editing frequencies and had synergistic effects with e-scaffold gRNAs without altering base editing windows across five target sites: (a) *ANGPT*, (b) *SERPINC1*, (c) *EPAS1*-1, (d) *TFPI*, and (e) *HIF1A*-2. The position of each nucleotide was numbered relative to the starting position of the target spacer sequences. All experiments were conducted in biologically independent triplicates. Error bars indicate standard error of the mean.

**Supplementary Table 1. List of target sequences and PCR primers used in this study.**

| gRNA name | Target sequences (5' to 3') | PAM | PCR-F | PCR-R |
|---|---|---|---|---|
| SERPINC1 | GAGGTTACAGTTCCTATCACAT | TGGAATAC | GGTGTTGGAGGTCATTCCTG | GTCCTCTGGAACCTCTGC |
| TFPI | GGCAGGGGCAAGATTAAGCAGC | AGGCATAC | TGGGTTCTGTATTTCAGAGATGA | TTCCCTCCACAATGGAAAAC |
| EPAS1-1 | GCAAGTTCATGGGACTTACACA | GGTGACAC | AAGCCTTGGAGGGTTTCATT | GTGGCTAGCACCTTCCACTC |
| ANGPT2 | GCTGTGCAGAGGGACGCGCCGC | TCGAATAC | ATGGGTCCTGCAGCTACACT | TTCCATGATGTTCTCCAGCA |
| EPAS1-2 | CAGGACAGCAGGGGCTCCTTGT | AGCCACAC | GGGCCAGGTGAAAGTCTACA | TCTCTGCCCACAGCATACAC |
| HIF1A-1 | TGAAACTCAAGCAACTGTCATA | TATAACAC | GGACAAGTCACCACAGGACA | TCCCCAAAAATGTTTATTTCTCA |
| HIF1A-2 | GCTATTCACCAAAGTTGAATCA | GAAGATAC | CCCTTCCCCTCACTGTATCA | GGCCAGCAAAGTTAAAGCAT |
| HPD-1 | TTTTCCACCCGTAGTATGGGGA | CACCACAC | GGAAGTAGGGGGTCCATGA | ACGCATCTGGTTAGGGTCAG |
| HPD-2 | TTCCACCCGTAGTATGGGGACA | CCACACAC | GGAAGTAGGGGGTCCATGA | ACGCATCTGGTTAGGGTCAG |
| HPD-3 | CCACCCGTAGTATGGGGACACC | ACACACAC | GGAAGTAGGGGGTCCATGA | ACGCATCTGGTTAGGGTCAG |
| HPD-4 | TCCACCAGGGTGTGTGTGGTGT | CCCCATAC | GGAAGTAGGGGGTCCATGA | ACGCATCTGGTTAGGGTCAG |
| AAVS1-2 | GAGTAGAGGCGGCCACGACCTG | GTGAACAC | AGAGCAGGGCCTTAGGGAAG | CGTGCGTCAGTTTTACCTGT |
| AAVS1-8 | GGAGTGTGACAGCCTGGGGCCC | AGGCACAC | GCAGATACTCTGCAGGAACG | CCCAAGTTTTGGACCCCTAA |
| HPD-2-M1-1 | TTCCACCCGTAGTATGGGGACg | CCACACAC | GGAAGTAGGGGGTCCATGA | ACGCATCTGGTTAGGGTCAG |
| HPD-2-M1-2 | TTCCACCCGTAGTATGGGGgCA | CCACACAC | GGAAGTAGGGGGTCCATGA | ACGCATCTGGTTAGGGTCAG |
| HPD-2-M1-3 | TTCCACCCGTAGTATGGaGACA | CCACACAC | GGAAGTAGGGGGTCCATGA | ACGCATCTGGTTAGGGTCAG |
| HPD-2-M1-4 | TTCCACCCGTAGTATaGGGACA | CCACACAC | GGAAGTAGGGGGTCCATGA | ACGCATCTGGTTAGGGTCAG |
| HPD-2-M1-5 | TTCCACCCGTAGTgTGGGGACA | CCACACAC | GGAAGTAGGGGGTCCATGA | ACGCATCTGGTTAGGGTCAG |
| HPD-2-M1-6 | TTCCACCCGTAaTATGGGGACA | CCACACAC | GGAAGTAGGGGGTCCATGA | ACGCATCTGGTTAGGGTCAG |
| HPD-2-M1-7 | TTCCACCCGcAGTATGGGGACA | CCACACAC | GGAAGTAGGGGGTCCATGA | ACGCATCTGGTTAGGGTCAG |
| HPD-2-M1-8 | TTCCACCtGTAGTATGGGGACA | CCACACAC | GGAAGTAGGGGGTCCATGA | ACGCATCTGGTTAGGGTCAG |
| HPD-2-M1-9 | TTCCAtCCGTAGTATGGGGACA | CCACACAC | GGAAGTAGGGGGTCCATGA | ACGCATCTGGTTAGGGTCAG |
| HPD-2-M1-10 | TTCtACCCGTAGTATGGGGACA | CCACACAC | GGAAGTAGGGGGTCCATGA | ACGCATCTGGTTAGGGTCAG |
| HPD-2-M1-11 | TcCCACCCGTAGTATGGGGACA | CCACACAC | GGAAGTAGGGGGTCCATGA | ACGCATCTGGTTAGGGTCAG |
| HPD-2-M2-1 | TTCCACCCGTAGTATGGGGAtg | CCACACAC | GGAAGTAGGGGGTCCATGA | ACGCATCTGGTTAGGGTCAG |
| HPD-2-M2-2 | TTCCACCCGTAGTATGGGagCA | CCACACAC | GGAAGTAGGGGGTCCATGA | ACGCATCTGGTTAGGGTCAG |
| HPD-2-M2-3 | TTCCACCCGTAGTATGaaGACA | CCACACAC | GGAAGTAGGGGGTCCATGA | ACGCATCTGGTTAGGGTCAG |
| HPD-2-M2-4 | TTCCACCCGTAGTAcaGGGACA | CCACACAC | GGAAGTAGGGGGTCCATGA | ACGCATCTGGTTAGGGTCAG |
| HPD-2-M2-5 | TTCCACCCGTAGcgTGGGGACA | CCACACAC | GGAAGTAGGGGGTCCATGA | ACGCATCTGGTTAGGGTCAG |
| HPD-2-M2-6 | TTCCACCCGTgaTATGGGGACA | CCACACAC | GGAAGTAGGGGGTCCATGA | ACGCATCTGGTTAGGGTCAG |
| HPD-2-M2-7 | TTCCACCCacAGTATGGGGACA | CCACACAC | GGAAGTAGGGGGTCCATGA | ACGCATCTGGTTAGGGTCAG |
| HPD-2-M2-8 | TTCCACttGTAGTATGGGGACA | CCACACAC | GGAAGTAGGGGGTCCATGA | ACGCATCTGGTTAGGGTCAG |
| HPD-2-M2-9 | TTCCgtCCGTAGTATGGGGACA | CCACACAC | GGAAGTAGGGGGTCCATGA | ACGCATCTGGTTAGGGTCAG |
| HPD-2-M2-10 | TTttACCCGTAGTATGGGGACA | CCACACAC | GGAAGTAGGGGGTCCATGA | ACGCATCTGGTTAGGGTCAG |
| HPD-2-M2-11 | ccCCACCCGTAGTATGGGGACA | CCACACAC | GGAAGTAGGGGGTCCATGA | ACGCATCTGGTTAGGGTCAG |

**Supplementary Table 2. List of potential off-target sites of cjABE target sites in the human genome.**

| gRNA name | | Target sequences (5' to 3') | PAM | Mismatches (bp) |
|---|---|---|---|---|
| ANGPT2 | ON | GCTGTGCAGAGGGACGCGCCGC | TCGAATAC | N/A |
| | OT1 | tCTGTGaAGAGGtACaCGCCGC | CCGAGTAC | 4 |
| EPAS1-1 | ON | CAGGACAGCAGGGGCTCCTTGT | AGCCACAC | N/A |
| | OT1 | aAGGAgAGCtGGGGCaCCTTGT | AATCATAC | 4 |
| HIF1A-1 | ON | TGAAACTCAAGCAACTGTCATA | TATAACAC | N/A |
| | OT1 | TaAAACaCAAGCAAaTGTgATA | AGAGATAC | 4 |
| HPD-1 | ON | TTTTCCACCCGTAGTATGGGGA | CACCACAC | N/A |
| | OT1 | TaTgtCgCCCGTAGTATGGGGA | AACAGCAC | 4 |
| AAVS1-8 | ON | GGAGTGTGACAGCCTGGGGCCC | AGGCACAC | N/A |
| | OT1 | tGAGTGTGACtGCCTGGGGCCC | AGTCACAC | 2 |
